# CRISPR‐mediated knockout of VEGFR2/KDR inhibits cell growth in a squamous thyroid cancer cell line

**DOI:** 10.1002/2211-5463.13399

**Published:** 2022-04-08

**Authors:** Ming‐Lin Tsai, Chia‐Hwa Lee, Li‐Chi Huang, Yu‐Hsin Chen, Wei‐Ni Liu, Chun‐Yu Lin, Kai‐Wen Hsu, Ai‐Wei Lee, Ching‐Ling Lin

**Affiliations:** ^1^ Department of General Surgery Cathay General Hospital Taipei Taiwan; ^2^ School of Medical Laboratory Science and Biotechnology College of Medical Science and Technology Taipei Medical University Taiwan; ^3^ TMU Research Center of Cancer Translational Medicine Taipei Medical University Taiwan; ^4^ Ph.D. Program in Medicine Biotechnology College of Medicine Taipei Medical University Taiwan; ^5^ Department of Internal Medicine Cathay General Hospital Taipei Taiwan; ^6^ Department of Endocrinology and Metabolism Cathay General Hospital Taipei Taiwan; ^7^ Department of cytology Cathay General Hospital Taipei Taiwan; ^8^ Institute of Bioinformatics and Systems Biology National Yang Ming Chiao Tung University Hsinchu Taiwan; ^9^ Center for Intelligent Drug Systems and Smart Bio‐devices National Yang Ming Chiao Tung University Hsinchu Taiwan; ^10^ Institute of New Drug Development China Medical University Taichung City Taiwan; ^11^ Research Center for Cancer Biology China Medical University Taichung City Taiwan; ^12^ Department of Anatomy and Cell Biology School of Medicine College of Medicine Taipei Medical University Taiwan; ^13^ Department of Internal Medicine School of Medicine College of Medicine Taipei Medical University Taiwan; ^14^ School of Medicine National Tsing Hua University Hsinchu Taiwan

**Keywords:** advanced thyroid cancer, CRISPR/Cas9, gene editing, receptor tyrosine kinase inhibitor, target therapy, VEGFR2/KDR

## Abstract

Squamous and anaplastic thyroid cancers are the most aggressive and life‐threatening cancer types in humans, with the involvement of lymph nodes in 59% of cases and distant metastases in 26% of cases of all thyroid cancers. The median survival of squamous thyroid cancer patients is < 8 months and therefore is of high clinical concern. Here, we show that both VEGFC and VEGFR2/KDR are overexpressed in thyroid cancers, indicating that VEGF/VEGFR signaling plays a carcinogenic role in thyroid cancer development. Using CRISPR/Cas9, we established a KDR knockout (KO) SW579 squamous thyroid cancer cell line that exhibited dramatically decreased colony formation and invasion abilities (30% and 60% reduction, respectively) when compared to scrambled control cells. To validate the potential of KDR as a therapeutic target for thyroid cancers, we used the KDR RTK inhibitor sunitinib. Protein analysis and live/dead assay were performed to demonstrate that sunitinib significantly inhibited cell growth signal transduction and induced cell apoptosis of SW579 cells. These results suggest that selective targeting of KDR may have potential for development into novel anti‐cancer therapies to suppress VEGF/VEGFR‐mediated cancer development in patients with clinical advanced thyroid cancer.

## Graphical abstract

The present study shows that VEGFR2/KDR overexpression in advanced thyroid cancer maintains cancer malignancy and cell survival. CRISPR/Cas9 gene editing as well as use of RTK inhibitor sunitinib of VEGFR2/KDR significantly disrupted thyroid cell cycle regulation, cell growth, and downstream signal transduction, resulting in decreased colony formation and invasion abilities. Thus, selective targeting of VEGFR2/KDR signaling may be a potential strategy for advanced thyroid cancer treatment.

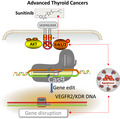

**Abbreviations**
ATC: anaplastic thyroid cancer
CCLE: cancer cell line encyclopedia
CRISPR/Cas9: clustered regularly interspaced short palindromic repeat/ CRISPR associated protein 9
KO: knockout
MAPK: mitogen‐activated protein kinase
PI3K: phosphoinositide 3 kinase
RTK: receptor tyrosine kinase
SKP2: S‐Phase kinase associated protein 2
STC: squamous thyroid cancers
TIDE: tracking of indels by decomposition
TKI: receptor tyrosine kinase inhibitor
VEGFR: vascular endothelial growth factor

Angiogenesis has been a long‐term concern as a critical step in blood vessel formation and cancer development. It is also involved in other biological activities in various diseases, including rheumatoid arthritis and diabetic retinopathy [1]. Among all the identified molecules associated with blood vessel formation, vascular endothelial growth factors (VEGFs) and vascular endothelial growth factor receptors (VEGFRs) proteins, and their pathway is definitely the most critical signal transduction in tumor angiogenesis. VEGFs are a group of heparin‐binding homodimeric glycoproteins that act via endothelial‐specific receptor tyrosine kinases. VEGFA overexpression is commonly found in the majority of solid tumors; therefore, VEGFA is the dominant target of antiangiogenic drugs [2]. The two polypeptide isoforms of VEGFB, VEGFB^167^, and VEGFB^186^ [3], interact with vascular endothelial growth factor receptor 1 (VEGFR1/FLT1) and activate downstream VEGFR signals [4]. Other VEGF‐associated molecules, such as VEGFC and VEGFD, alternatively bind and activate vascular endothelial growth factor receptor 2 (VEGFR2/KDR) and 3 (VEGFR3/FLT4) [5]. In addition, angiopoietin receptors such as Tie‐1 and Tie‐2/Tek contribute to crucial human blood vessel formation in endothelial cells [6].

KDR is the predominant mediator of VEGF‐induced angiogenesis, and its blockade has been widely investigated. Several pharmacological research and clinical studies indicate that the KDR blockage would be a promising strategy to suppress tumor‐induced angiogenesis [7]. In particular, small molecules such as sorafenib, sunitinib, and pazopanib multiple RTK inhibitors have been effectively applied in the clinic to prevent cancer development and metastasis. Accordingly, KDR expression in cancer cells has been extensively observed in various human malignancies, including breast cancer [8], lung cancer [9], glioblastoma [10], gastrointestinal cancer [11], hepatocellular carcinoma, renal cell carcinoma [12], and ovarian cancer [13]. However, the expression evaluation and the biological function of KDR in advanced thyroid cancer remain unclear.

The VEGF/VEGFR signaling has been well‐investigated in cancer proliferation and survival during tumorigenesis. A number of phosphorylated sites, such as Y801, Y951, Y1175, and Y1214, in the VEGFR2/KDR intracellular domains mediate critical signal transductions, including PI3K/AKT, MAPK, and PKC pathways [14]. The activated second messengers of PI3K/AKT and MAPK molecules effect numerous and diverse biological effects by phosphorylating downstream proteins, which include AKT (protein kinase B), mTOR (mammalian target of rapamycin), ERK1/2 (extracellular signal‐regulated kinase 1/2), FAK (focal adhesion kinase), and p70S6K (ribosomal protein S6 kinase), and therefore manipulating cellular metabolism, protein synthesis, apoptosis pathways, transcription factor regulation, and cell cycle regulation [15, 16].

Thyroid cancer is relatively rare among all cancer types; it is especially commonly diagnosed at a younger age rather than in elderly adult patients. Statistically, females are over three times more likely to develop thyroid cancer than males. Thyroid cancer is usually generally favorable and is often cured with surgery, and if indicated, radioactive iodine is an ideal treatment for papillary and follicular well‐differentiated thyroid cancers (DTC). Despite the average thyroid cancer survival rate being around 95% at 40 years, rare and aggressive poor‐differentiated thyroid cancers are extremely severe and life‐threatening. In addition, these advanced thyroid cancers, such as anaplastic thyroid cancer (ATC) and squamous thyroid cancers (STC) cancers are often resistant to radioactive iodine treatment, and the disease often exhibits local recurrence or distant metastasis when thyroid cancer is eventually diagnosed [17]. Sorafenib (Nexavar) was the first‐line FDA‐approved treatment for progressive radioiodine‐refractory DTC but was then replaced by lenvatinib (Lenvima) in 2015 [18]. Recently, FDA approved VEGF inhibition therapy such as cabozantinib, vandetanib, and their combination for medullary thyroid cancer patients [19, 20]. The results indicated that cabozantinib/vandetanib or the combo treatments improved patient survival; however, adverse effects of these drugs could be debilitating [21]. Even though targeted therapies for papillary, follicular, and medullary thyroid cancers are increasingly available, targeted medication is yet to be developed for ATC and STC. Thus, developing a target therapy for advanced ATC and STC cancer types is essential.

In the current study, we investigated the oncogenic roles of KDR expression in squamous advanced thyroid cancer cells. CRISPR‐mediated *KDR* gene editing would enable the investigation of the biological impact of KDR expression on cell survival and cancer metastasis in thyroid cancers. Furthermore, it is highly possible that targeting KDR would provide effective anti‐cancer therapy for squamous thyroid cancer, ultimately improving the disease survival rate of advanced thyroid cancer patients in the clinic.

## Materials and methods

### Squamous thyroid cancer culture

The human squamous thyroid SW579 cancer cell line was purchased from Bioresource Collection and Research Center (BCRC), Hsinchu, Taiwan. For regular cell culture, SW579 cell was maintained in DMEM/F‐12 culture medium (Gibco, Carlsbad, CA, USA), supplemented with 10% (v/v) fetal bovine serum (FBS, Biological Industries, Kibbutz Beit Kaemek, Israel), 100 units·mL^−1^ penicillin, and 100 mg·mL^−1^ streptomycin at 37 °C with 5.0% CO_2_ condition. The medium was replaced every 2 days, and when cells reached 80% confluence, they were passaged using 0.25% trypsin/EDTA (Gibco).

### Gene expression analysis

Gene expression profile is obtained from the Cancer Cell Line Encyclopedia (CCLE‐ https://cancergenome.nih.gov/), whereas the disease survival rate was analyzed through the public database of The Human Protein Atlas (https://www.proteinatlas.org/).

### Immunohistochemistry

KDR protein expressions were immunostained by VEGFR2 primary antibody (#2479) from Cell Signaling Technology (Danvers, MA, USA) in human Papillary thyroid cancer tissues (Patient ID. 2623) and normal thyroid gland tissues (Patient ID. 1501). The KDR protein expression on tissue array images were obtained from Human Protein Atlas.

### Plasmid construction and lentiviral production

Lentiviral particles production was performed previously [22, 23]. In brief, the lentiCRISPR V2 containing *KDR*_sgRNAs was co‐transfected with pMD2.G and psPAX2. Lentiviral containing medium was collected at 36 and 72 h and quantified by Q‐PCR analysis.

### Designing *KDR* on‐target and off‐target sgRNAs

Custom *KDR* protospacers were designed using the E‐CRISPR Design website (http://www.e‐crisp.org/E‐CRISP/) through *KDR* (NM_002253.3) sequence input. The highest score sgRNAs were selected for the *KDR* gene target, whereas the corresponding off‐target sequences were examined for potential off‐target gene editing.

### Sanger sequencing and gene editing efficiency assay

*KDR* gene‐edited SW579 cells were harvested, and the genomic DNA was extracted for DNA sequencing. Following primers were used to perform PCR amplification: *KDR* forward primer: TCTTGGCAAACAGCCTTTAG and *KDR* reverse primer: TCACAGTCTGGCACTTCATA. Off‐target sequences were PCR‐amplified using the following primers: ABCA4, forward ATGTAAACAAACAAACAAGACGGT and reverse AAGTGACTCGATATGTCCAGAATAG. The PCR products were purified and Sanger sequenced.

### RNA‐guided engineered nuclease‐restriction fragment length polymorphism (RGEN‐RFLP) assay

PCR amplification products of the *KDR* DNA locus were used by following primers: *KDR* DNA sense primer: GCGGCCTCTAATACGACTCACTATAGGGTTCCCGGTAGAAGCACTTGTGTTTTAGAG and *KDR* DNA reverse primer: AAAAAAGCACCGACTCGGTGCCACTTT‐TTCAAGTT. The PCR products were collected, purified, and incubated with Cas9 protein and *KDR* sgRNAs. RNase A and proteinase K were added to remove the retained RNA and Cas9 protein. The products were then analyzed by agarose gel electrophoresis.

### MTT cell viability assay

SW579 cancer cell viability was measured by MTT assay [24]. SW579 cells were plated in a 96‐multi‐well plate overnight and had indicated sunitinib concentration treatment. The drug‐contain medium was removed and fresh culture medium containing 1 μg·mL^−1^ of MTT was added and incubated for two hours. DMSO was added to resolve formazan and to determine the absorbance at OD 570 and 630 nm.

### Colony formation assay

The SC and *KDR* gene edit SW579 cells were plated in 6‐well tissue culture plates at a density of 500 cells·mL^−1^, 3 mL·well^−1^, waiting 28 days incubation to allow colonies to form. The colonies were fixed with 4% formaldehyde, stained with 0.5% crystal violet for two hours, washed to remove the excess dye, and photographed. The colony numbers were counted by image J software (Version 1.52a, NIH, Bethesda, MD, USA).

### Protein extraction, western blotting, and antibodies

SW579 protein determination was resolved by sodium dodecyl sulfate‐polyacrylamide gel electrophoresis (SDS/PAGE). The primary antibodies were purchased from the following vendors: the anti‐AKT (GTX128414), anti‐P21 (GTX629543), and anti‐P27 (GTX100446) antibodies were from GeneTex International Corporation (Hsinchu, Taiwan). The anti‐SKP1 (32‐3800) and anti‐SKP2 (32‐3300) were from Invitrogen (Thermo Fisher Scientific, Waltham, MA, USA). The anti‐GAPDH (sc‐32233), anti‐p‐ERK (sc‐7383), and anti‐P53 (sc‐126) antibodies from Santa Cruz Biotechnology (Santa Cruz, CA, USA). The anti‐c‐Caspase 3 (#9664) and anti‐VEGFR2 (#3174) antibodies were from Cell Signaling Technology. All western blots were repeated at least three times independently.

### Live/dead cell viability assay

Cells were seeded in 12‐well plates overnight and incubated with imatinib for 24 h in standard culture conditions. The medium was removed and the cells treated for 30 min in the dark with 1 m calcein‐AM and 10 m propidium iodide (PI) prepared in a standard culture medium. The live fluorescence images were captured under a light wavelength of 488 nm (green emission) to show viable cells. The same image of the cells was also excited with a light of wavelength 532 nm (red emission) to show the dead cells. Five random photos were taken in each experimental group, and the percentage of cell death/live cell was estimated.

### Statistical analysis

All statistical comparisons are presented as the mean ± standard error. The different significance was determined with one‐way ANOVA among dose‐dependent drug treatment, and then Student’s t‐test was used to estimate the significance between groups. The figures were generated by SigmaPlot (San Jose, CA, USA). A *P*‐value < 0.05 was considered statistically significant, and all statistical tests were two‐sided.

## Result

### Revealing VEGF/VEGFR subunit expression in thyroid cancers

**Fig. 1:**
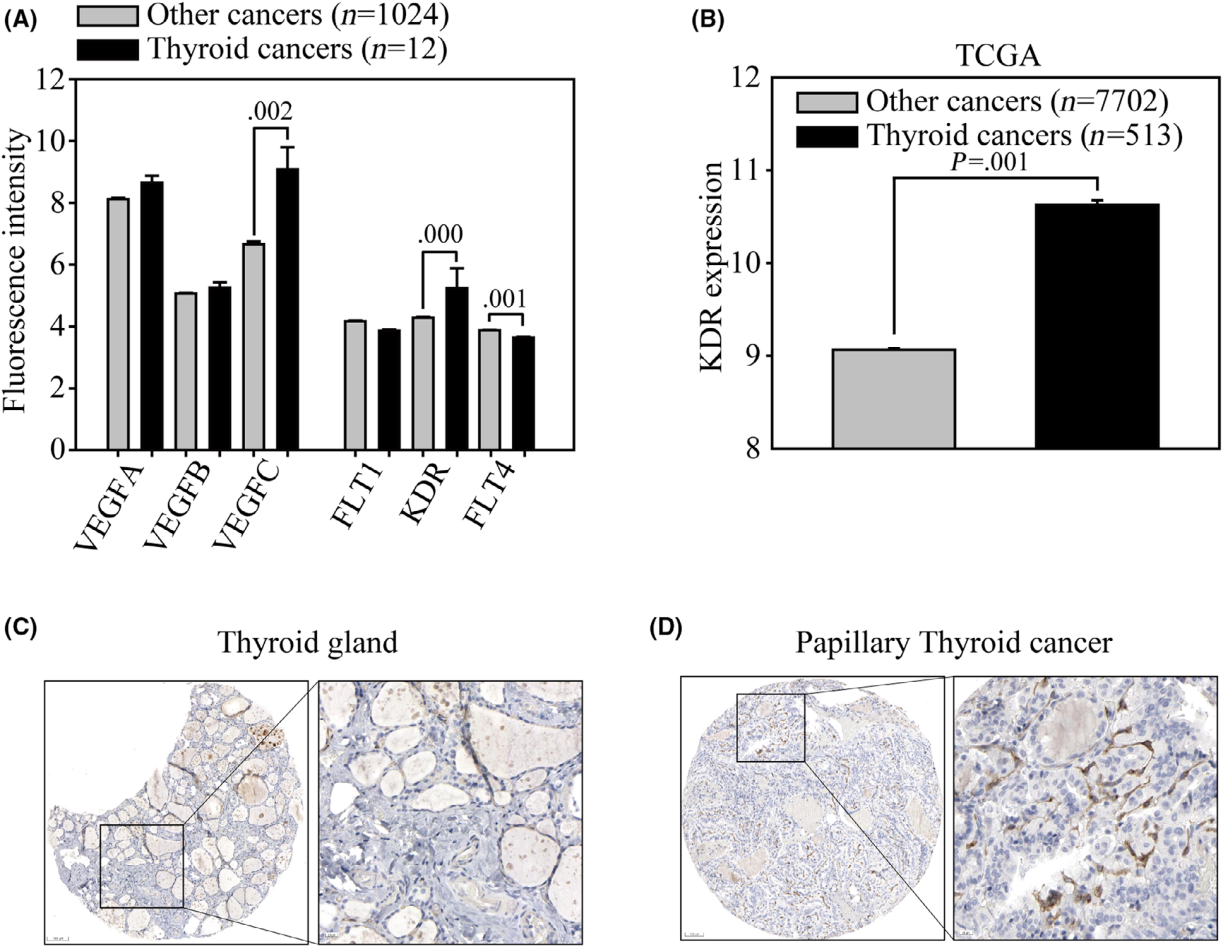
Revealing VEGF/VEGFR expressions in human thyroid cancers. (A) VEGF/VEGFR gene expression were analyzed in the CCLE microarray database. All the VEGF/VEGFR gene expressions were divided into thyroid (12 cells, black bars) and other cancer types (1024 cancer cells, gray bars). The average fluorescence intensity of VEGF ligands (VEGFA, VEGFB, and VEGFC) and the VEGF receptors (VEGFR1/FLT1), VEGFR2/KDR and VEGFR3/FLT4) were subgrouped and analyzed. (B) Determination of *KDR* expression using the TCGA database in all 8215 cancer cells. *KDR* expression was determined in thyroid tissue cancers (*n* = 513, black bars) and cancers in other tissues (*n* = 7702, gray bars). Gene expression is illustrated as the mean ± standard error of the fluorescence intensity. The significance of gene expression was determined with *t*‐test. KDR protein expression in (C) normal human thyroid gland and (D) papillary cancer tissues were obtained from the Human Protein Atlas. The images are presented at low (left) and high magnification (right). Scale bar = 100 µm.

To identify all VEGF/VEGFR subunits expression among human malignancies, we collected and analyzed three VEGF ligands (VEGFA, VEGFB, and VEGFC) and three VEGF receptors (VEGFR1/FLT1, VEGFR2/KDR, and VEGFR3/FLT4) gene expression profiles in 1024 human cancers caused by 24 tumor types in the Cancer Cell Line Encyclopedia (CCLE). Comparing VEGF/VEGFR subunit expressions among thyroid cancer and other cancer types, the result clearly showed that the mean log^2^ fluorescence intensity of VEGFC was 9.08 ± 0.71 in thyroid cancers and 6.66 ± 0.084 in other cancer types (5.33‐fold, *P* = 0.002). In contrast, the *KDR* gene expression was 5.24 ± 0.64 in thyroid cancers and 4.28 ± 0.024 in other cancers (1.94‐fold, *P* = 0.001), respectively (Fig. 1A). In addition, although FLT4 obtains a minor elevated expression in other cancer types than in thyroid cancers, the general FLT4 expression in all cancer types is weakly determined among all VEGF/VEGFR subunits. To confirm the predominant *KDR* expression in thyroid cancers, we further analyzed *KDR* gene expression in both thyroid and other types of cancers through the TCGA database (Fig. 1B). Five hundred thirteen thyroid cancer tissues had significantly higher *KDR* gene expression intensity (10.63.74 ± 0.049) than 7702 other types of cancers (9.06 ± 0.018) (*P* < 0.001). Next, we investigated whether KDR protein expression is highly expressed in clinical thyroid cancer tissues through The Human Protein Atlas [25]. In tissue microarray, the immunohistochemistry assay showed weak KDR protein expression (Fig. 1C), whereas thyroid cancer cells exhibited strong but non‐homogeneous KDR expression (arrowhead, Fig. 1D). The above evidence implies that thyroid cancer is a heterogeneous cancer type, and the KDR‐expressing cell subpopulation may drive cancer growth, disease progression, therapy resistance [26], or even distant metastasis [27].

### CRISPR/Cas9 based *KDR* gene edit on human thyroid cancer

**Fig. 2:**
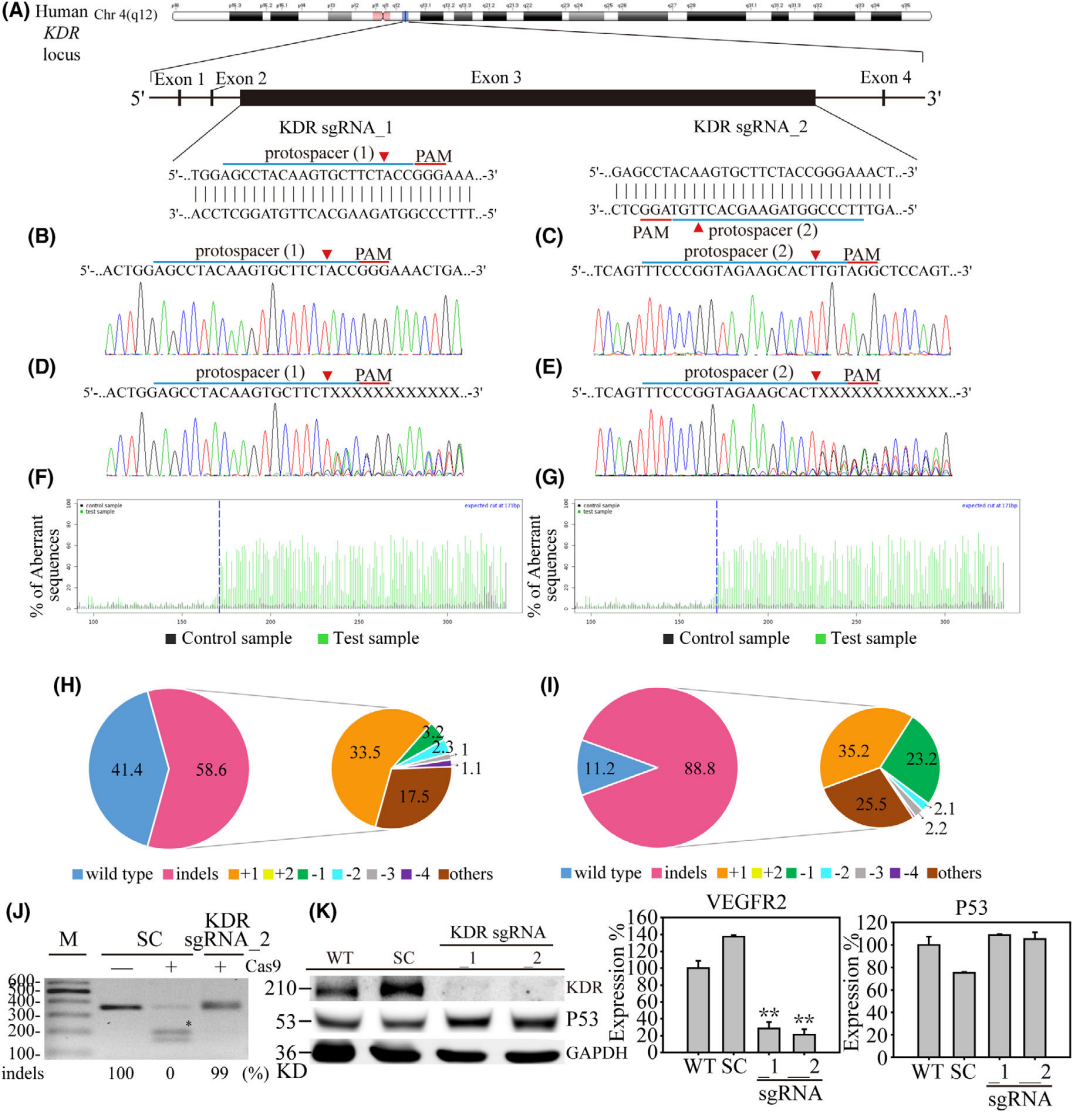
CRISPR/Cas9‐based *KDR* gene editing in SW579 cells. (A) Schematic representation of the human *KDR* gene locus. Two protospacer sequences (blue underlining) of the *KDR* gene locus were selected for CRISPR/Cas9 gene editing, whereas the predicted Cas9 cleavage site is arrowheaded. SW579 thyroid cancer cells were transfected with scrambled (SC) and *KDR* sgRNAs containing lentivirus for three days, followed by 2 days of puromycin selection. The DNA extracts from these cells were then Sanger sequenced for *KDR* protospacer (1) and protospacer (2) gene locus. (B) and (C) show the wildtype *KDR* sequences in SW579 cells, whereas (D) *KDR* sgRNA_1 and (E) *KDR* sgRNA_2 produced multiplex DNA mixtures. TIDE algorithm analysis confirmed that (F) *KDR* sgRNA_1 and (G) *KDR* sgRNA_2 specifically cleaved gene edit sites of SW579 cells. The pie chart shows the sgRNAs caused gene indels (insertions or deletions) in the cell pool. The gene editing efficiency of (H) *KDR* sgRNA_1 and (I) *KDR* sgRNA_2 is represented in pink, whereas the two most common indels are represented in indicated colors. (J) RGEN‐RFLP assay was then performed to confirm the gene edit efficiency. The PCR product of the *KDR* protospacer (2) locus was digested with *KDR* sgRNA_2 and Cas9 addition. The fragments of the cleaved DNA are highlighted (asterisk). (K) Protein analysis and quantified result determined KDR protein expression in wildtype (WT), scrambled (SC), *KDR* sgRNA_1, and *KDR* sgRNA_2 SW579 cells by western‐blotting assay. Data are presented as the mean protein expression ± standard error and analyzed with Student's *t*‐test. *P*‐values are two‐sided; *P*‐values ≦ 0.05 are represented by an asterisk, whereas *P*‐values ≦ 0.01 are represented by two asterisks. GAPDH serves as an internal loading control.

In the last decade, gene editing such as CRISPR/Cas9 based technology has been widely used to investigate the biological function of genes. The current study used CRISPR/Cas9 engineering in a lentivirus delivery system to target the *KDR* DNA locus of human chromosome 4 (q12) on SW579 thyroid cancer (Fig. 2A). Two protospacers of *KDR* sgRNA_1 and sgRNA_2 were designed through E‐CRISPR (http://www.e‐crisp.org/E‐CRISP/). It can be easily identified on the *KDR* genomic map that *KDR* sgRNA_1 targets protospacer (1) DNA site and sgRNA two targets protospacer (2) on both the plus‐ and negative‐strand of *KDR* exon3 DNA locus. Through optimized lentivirus transfection protocol [28], SW579 thyroid cancer was exposed in SC (scrambled), *KDR* sgRNA_1, *KDR* sgRNA_2 virus for three days, and the antibiotic selected for 48 h. Through the Sanger sequence, both *KDR* protospacer (1) and protospacer (2) locus showed homogeneous and wildtype DNA alleles in SC virus exposed SW579 cells (Fig. 2B,C). On the other hand, *KDR* sgRNA_1 and *KDR* sgRNA_2 virus exposed that SW579 cells obtained significant DNA disruptions (Fig. 2D,E) on predicted Cas9 cleavage sites (3‐4 nucleotide above PAM sequence, red arrowhead) (Fig. 2F,G). We next used tracking of indels by decomposition (TIDE) to reveal the nucleotide insertion and deletion (indels) mutations of the *KDR* gene locus. The pie‐chart showed that *KDR* sgRNA_1 caused a total of 58.6% gene disruption, which consisted of the most frequent 1‐bp insertion (+1) and other mutations with 33.5% and 17.5% gene editing in *the KDR* sgRNA_1 virus‐infected SW579 cell pool (Fig. 2H). *KDR* sgRNA_2, however, caused a significant 88.85% gene disruption, consisting of the most frequent 1‐bp insertion (+1), other mutations, and 1‐bp deletion (−1) with 33.5%, 25.5%, and 23.2% gene editing in *KDR* sgRNA_2 virus‐infected SW579 cell pool (Fig. 2I). Furthermore, we applied RGEN‐RFLP to confirm the *KDR* gene editing efficiency. The gel electrophoresis showed that the protospacer (2) DNA PCR product of SC transfected cells was 360 base pairs (uncut) (Fig. 2J). However, when *KDR* sgRNA_2 and Cas9 were present, the protospacer (2) DNA PCR product from SC was digested at 200 and 160 bps DNA fragments (with an asterisk), indicating a 100% wildtype DNA in the input sample. On the other hand, the protospacer (2) DNA PCR product from *KDR* sgRNA_2 virus‐infected cells showed 99% at 360 bps (uncut), confirming the high gene editing efficiency in *KDR* sgRNA_2 virus transfected SW579 thyroid cancer cells. Next, we used western blot to quantify KDR protein expression of the above sgRNA virus‐infected cells. The blotting demonstrated that protein expression in both *KDR* sgRNA_1 and *KDR* sgRNA_2 cells were significantly abolished (*P* ≤ 0.01), compared to wildtype (WT) and SC cells (Fig. 2K, Fig. S1). In addition, P53, a central governor of cell cycle regulation, was not associated with KDR signal transduction. This data implies that P53 regulation may not be directly involved in KDR induced biological function changes.

### *KDR* gene editing inhibits cancer cell growth and invasion through cell cycle regulation in thyroid cancers

**Fig. 3:**
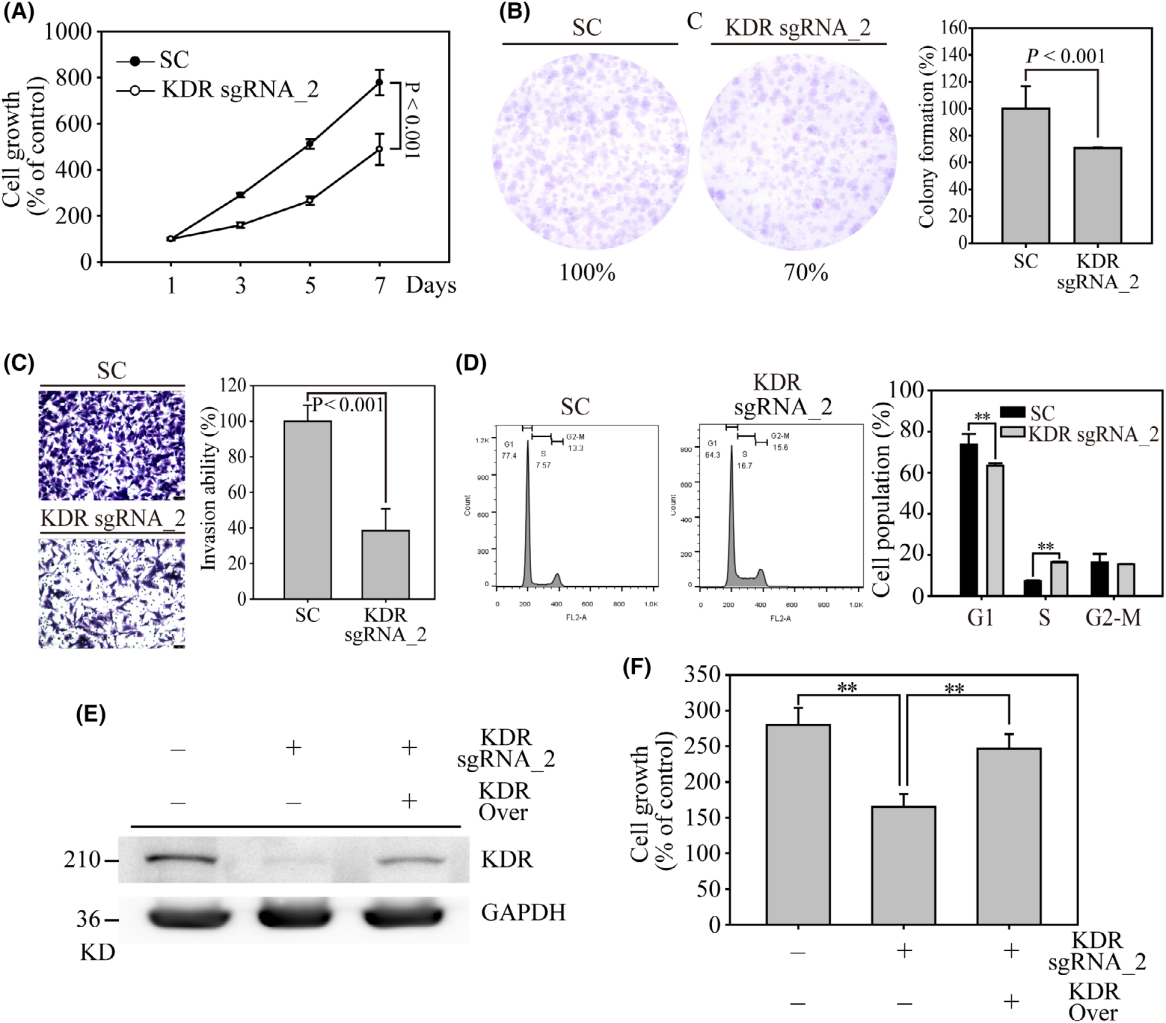
Biological evaluation of KDR in SW579 thyroid cancer cells. (A) MTT based cell growth determination was performed to analyze the cell proliferation on SC‐ and *KDR* sgRNA_2 SW579 cells. (B) Colony formation was used to access long‐term cell growth on SC‐ and *KDR* sgRNA_2 SW579 cells. The colony from these cell pools was stained by 1% crystal violet. The colony number was determined and calculated using imagej software. (C) The matrigel‐based invasion assay was performed to determine invasion alternation in *KDR* gene edit SW579 cells. Indicated cell numbers of SC‐ and sgRNA_2 transfected SW579 cells were cultured in transwell for three days. The transwell membranes were finally stained with 1% crystal violet, and the image was captured by 100× magnification. Scale bar = 100 µm. (D) Flow cytometry‐based cell cycle analysis on SC‐ and *KDR* sgRNA_2 SW579 cells. The numbers of G1, S, and G2‐M cells were measured and illustrated in the bar chart figures. (E) Rescue assay showed significant KDR protein expression on *KDR* sgRNA_2 SW579 cells by western blot analysis, whereas GAPDH served as internal control. (F) MTT assay on SC‐, *KDR* sgRNA_2, and KDR rescued cells were determined by a 24 h cell culture. The cell growth and colony formation were calculated as percentage of control cell numbers. Data are presented as the mean ± standard error and analyzed with Student's *t*‐test. *P*‐values are two‐sided; *P*‐values ≦ 0.05 are represented by an asterisk, whereas *P*‐values ≦ 0.01 are represented by two asterisks. All western blots were repeated at least three times, independently.

We further used *KDR* sgRNA_2 virus‐infected SW579 cells to investigate the potential biological impact of KDR in advanced thyroid cancers. First, we used MTT to determine whether cell proliferation was affected in *KDR* gene edited cells (Fig. 3A). During a seven days observation, we found that *KDR* sgRNA_2 cells obtained a significant 38% cell growth reduction (*P* < 0.001), compared to 100% cell growth rate in SC cells. In addition, by performing the colony formation assay, we discovered that *KDR* sgRNA_2 cells reduced to only 70% of colony numbers (*P* < 0.001), compared to SC cells (Fig. 3B, Fig. S2A). Next, we performed the matrigel‐coated transwell assays to examine the impact of the absence of *KDR* on advanced thyroid cancer (Fig. 3C, Fig. S2B). The microscopic images showed that *KDR* sgRNA_2 cells obtained a 62% significant reduction in invasion cell numbers, compared to 100% invasion ability in SC cells (*P* < 0.001). Next, we used flow cytometry to reveal KDR‐regulated cell cycle arrest (Fig. 3D). *KDR* sgRNA_2‐containing SW579 cells showed a significant decrease in the G1 phase cell population and an increase in the S‐phase population, whereas the G2‐M phase cell population was not affected. This observation confirmed the previous finding that the inhibition of KDR and STAT3 effectively increases S‐phase cell arrest and results in radiosensitizing effects in non‐small cell lung cancer cells [29]. Furthermore, we performed the gene rescue assay to confirm these bio‐functional alternation findings in *KDR* gene edited thyroid cancer cells (Fig. 3E, Fig. S2C). The western blot showed that the overexpression of the *KDR* gene in *KDR* sgRNA_2 cells significantly restored KDR protein, compared to *KDR* sgRNA_2 cells. Finally, cell growth assay showed that the loss of cell proliferation ability in *KDR* sgRNA_2 cells was remarkably recovered in *KDR* gene rescued cells, indicating the tremendous anti‐thyroid cancer potential of KDR targeting (Fig. 3F).

### Potential off‐target effects of custom‐designed *KDR* sgRNAs

**Fig. 4:**
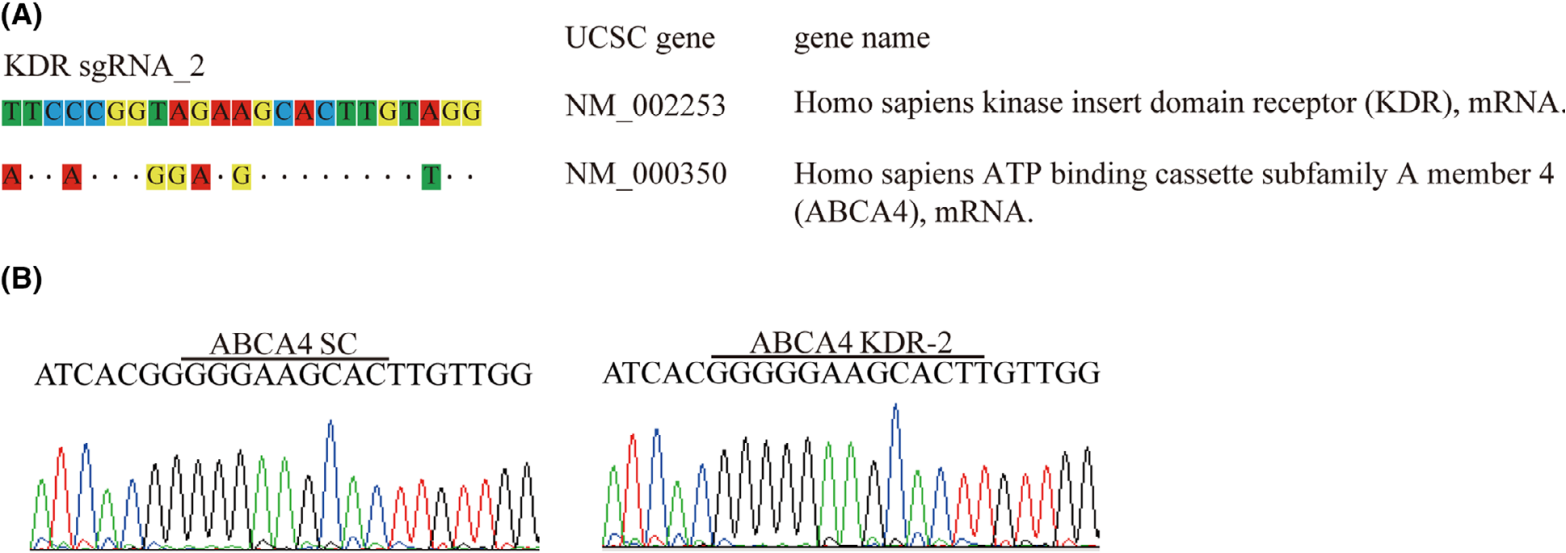
Investigating off‐target potential on *KDR‐*targeted SW579 cells. (A) Human ATP binding cassette subfamily A member 4 (ABCA4) is the most highly similar off‐target gene to the *KDR* sgRNA_2 sequence. The identical gene sequences on ABCA4 are represented by dots, whereas mismatch sequences are shown by nucleotide substitution. (B) Sanger sequence determination of SC‐ and *KDR* sgRNA_2 caused gene interruptions in the *KDR* protospacer (2) locus.

To clarify the off‐target potential, the *Homo sapiens* ATP binding cassette subfamily A member 4 (ABCA4) gene, was selected for potential off‐target evaluation. Through sequence comparison, it was found that the ABCA4 gene shares only six nucleotides mismatched to the *KDR* sgRNA_2 sequence (Fig. 4A). The Sanger sequencing data of ABCA4 illustrated homogenous wildtype *KDR* protospacer (2) sequence (Fig. 4B), indicating a specific gene targeting to *KDR* gene locus by *KDR* sgRNA_2 containing a virus. The above result provides strong evidence that KDR‐targeted therapy may effectively prevent thyroid cancer‐triggered cancer metastasis.

### Potential TKI for KDR targeted therapy on advanced thyroid cancer

**Fig. 5:**
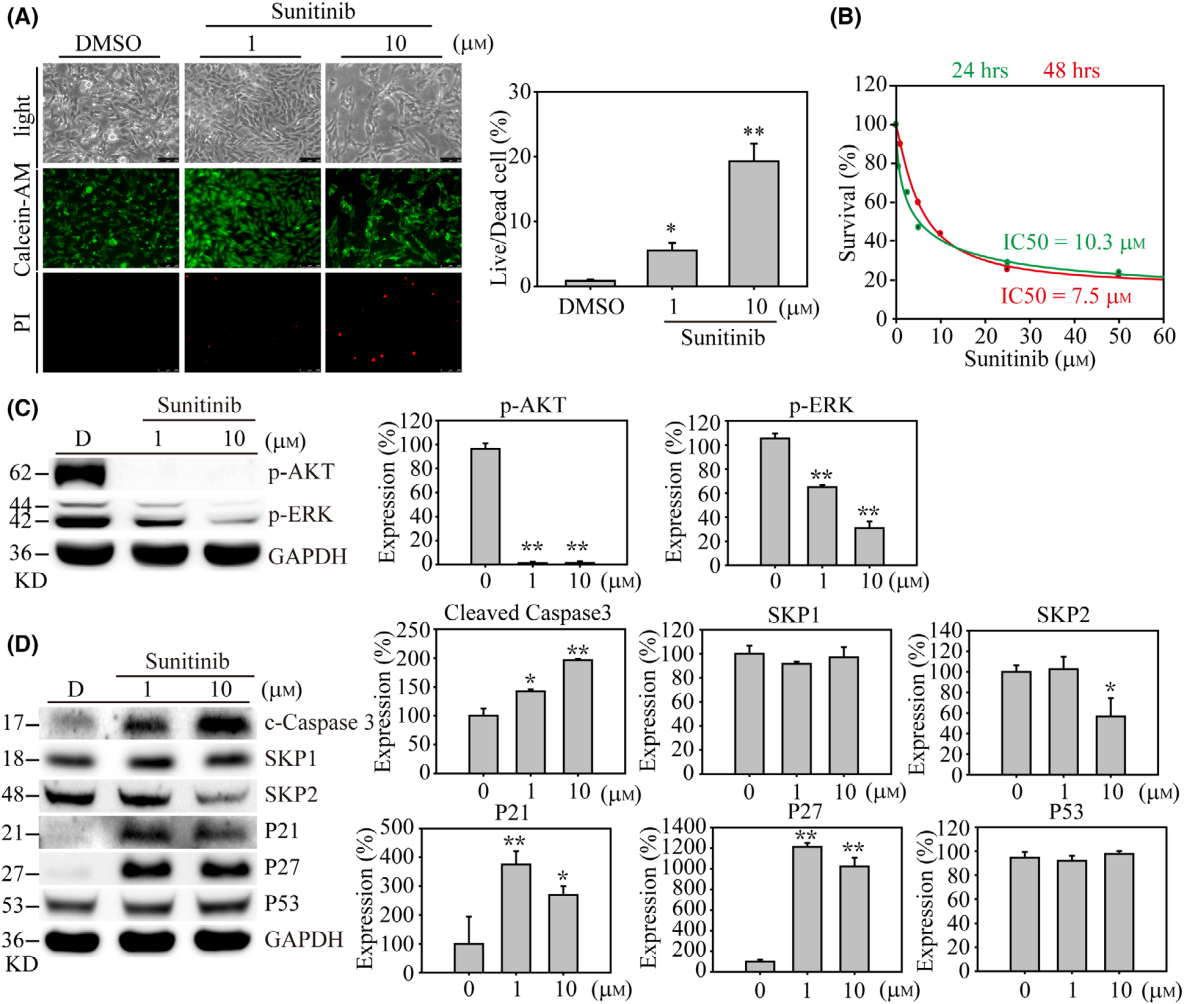
Sunitinib induced cell apoptosis and inhibited cell growth‐associated signaling in SW579 cells. (A) The live/dead‐based cell viability assay was determined with DMSO, 1 and 10 μm sunitinib exposures on SW579 cells. After 24 h, the calcein‐AM/PI (Propidium Iodide) mixture was added to the medium for 30 min. Five random images were taken in each experimental group. Live and dead cells of SW579 cells were determined by green and red fluorescents under 50× magnification, respectively. The percentage of cell live/dead analysis was illustrated. The scale bar = 250 µm. (B) The half‐maximal inhibitory concentration (IC50) of sunitinib was determined by MTT assay. The green curve represents 24 h of drug treatment, whereas the red curve represents 48 h. Western blotting and quantified results show (C) cell growth signaling, (D) cell cycle regulation, and apoptosis event associated protein alternations in sunitinib dose‐dependent exposure. Data are represented as the protein expression mean ± standard error and analyzed with one‐way ANOVA. In addition, Student's *t*‐test was performed to evaluate the significance between groups. *P*‐values are two‐sided; *P*‐values ≦ 0.05 are represented by an asterisk, whereas *P*‐values ≦ 0.01 are represented by two asterisks. All western blots were repeated at least three times, independently.

Several KDR‐included multiple tyrosine kinase inhibitors (TKIs) have been identified and FDA approved for anti‐cancer therapy, including sorafenib, sunitinib, regorafenibinib, and cabozantinib [2]. Among these potential KDR TKIs, sorafenib and sunitinib were pioneering drugs, resulting in the highly successful treatment of mRCC during drug development [30]. Subsequently, other agents emerged with similar modes of action but improved toxicity profiles. Accordingly, sunitinib has a low half‐maximal inhibitory concentration (IC50) of 80 nm against KDR in cancer cells [31]. In this study, we evaluated the anti‐cancer effects of sunitinib on SW579 cells to reveal whether or not KDR targeting could potentially be used as cancer therapy for advanced thyroid cancer patients. First, a live/dead assay of sunitinib‐induced cancer cell apoptosis was determined on SW579 cells (Fig. 5A, Fig. S3). The fluorescent images showed that SW579 cells demonstrated a significant 5.52 ± 1.16 and 19.29 ± 2.71% cell death in 1 and 10 μm sunitinib exposure groups, whereas DMSO control treatment obtained a minor 0.81 ± 0.21% dead cells. In addition, with sunitinib does‐dependent treatment on SW579 cells, the MTT assay determined that sunitinib had an IC50 of 10.3 μm for 24 h and 7.5 μm or 48 h drug incubation (Fig. 5B). Next, we investigated the biological impacts of sunitinib exposure on signal transduction and the cell cycle on SW579 cells. Western blot analysis illustrated that 1 and 10 μm sunitinib treatment significantly inhibited ERK and AKT phosphorylation in SW579 cells (*P* ≤ 0.01), compared to the DMSO control (Fig. 5C, Fig. 4A). Furthermore, thyroid cancer cells exposed to sunitinib dose dependent treatments showed significant Caspase 3 cleavage, P21, and P27 protein expressions, whereas P53 was not affected (Fig. 5D, Fig. 4B). This data confirms the previous finding in Fig. 2K, where it showed that P53 regulation was not associated with KDR signal transduction. Interestingly, levels of the well‐known oncogenic protein SKP2, which belongs to the F‐box family of substrate‐recognition subunits in SCF ubiquitin‐protein ligase complexes, were dramatically reduced under 10 μm sunitinib treatment, whereas SKP1 protein expression was not significantly altered. These results imply that sunitinib‐induced KDR inhibition significantly suppresses growth signaling and induces cell cycle arrest, mainly through the P53‐independent pathway, eventually causing cell death in advanced thyroid cancers.

## Discussion

Due to the treatment options for clinical aggressive and radioiodine‐refractory differentiated thyroid cancer patients being limited in the current clinic, chemotherapy such as doxorubicin may still be the most effective treatment for advanced thyroid cancer. Due to the treatment options for clinical aggressive and radioiodine‐refractory differentiated thyroid cancer patients being limited in the current clinic, chemotherapy such as doxorubicin is one of the few available treatments for advanced thyroid cancer, providing a better disease prognosis in thyroid cancer patients with local or distant metastasis. However, the satisfaction of using doxorubicin on metastatic differentiated thyroid cancer patients is disappointing, with a 37% partial response rate and 32% stable disease rate. Therefore, monotherapy may not effectively prevent cancer progression in aggressive thyroid cancers. In contrast, combination regimens that include different anti‐cancer agents may be a better anti‐cancer strategy for metastatic thyroid cancer patients.

A previous study illustrated that EGFA, KDR, and FLT4 are profoundly and distinctly expressed in thyroid glands [32]. Targeting VEGFRs and PDGFRs using small‐molecule tyrosine kinase inhibitors may therefore potentially cause thyroid function test abnormalities. However, the mechanism of VEGFRs underlying sunitinib‐ and sorafenib‐induced thyroid function test abnormalities are not fully understood. Many studies have investigated this mechanism with conflicting results. One study found out that 56 of 66 different types of cancer patients (85%) receiving sunitinib had at least one thyroid function test abnormality that was consistent with hypothyroidism [5]. In addition, 9 out of 17 of those patients had significant improvement of symptoms after thyroid hormone replacement. Another conflicting study indicated that receiving either sunitinib or sorafenib was not significantly related to the development of hypothyroidism [33]. The result of this investigation is consistent with another study that found that thyroid dysfunction did not occur more frequently in patients who received sunitinib or sorafenib treatments compared to those who did not [34]. Therefore, further research is needed to explain this controversial observation.

Skp2 is an extreme member of the F‐box family of substrate recognition subunits of SCF (Skp, Cullin, and F‐box) ubiquitin‐protein ligase complexes that have been discovered to be involved in the ubiquitin‐mediated degradation of several key regulators involved in cell cycle progression, including the cyclin‐dependent kinase inhibitor p27, which is a dosage‐dependent tumor suppressor protein. The SKP2 protein is oncogenic and overexpressed in several human malignancies, such as breast and colon cancers. Recently, a research team found that SKP2 is overexpressed in 45.5% of papillary thyroid cancers and that SKP2 expression is also significantly associated with extrathyroidal extension and distant metastasis [35]. In this study, the combination of bortezomib and TRAIL induced a synergistic apoptotic response and suppressed cancer/tumor growth of papillary thyroid cancers *in vitro* and in animal models, suggesting that SKP2 is a potential therapeutic target in aggressive papillary thyroid cancers. The present study found that sunitinib significantly decreased SKP2 protein expression and caused a dramatic increase in cell apoptosis in SW579 cells, whereas P27 protein expression was not affected. This unexpected discovery may be due to the P27 protein being regulated independently of SKP2 in the absence of CDK2 [36]. Therefore, CDK2 is a critical molecule that modulates the SKP2/P27 axis in human cancers.

In this study, CRISPR/Cas9‐mediated KDR gene interruption had a specific biological impact on the KDR receptor. In the present study, we examined cancer cell proliferation, cell cycle regulation, and, most importantly, the metastatic effect of KDR in SW579 squamous advanced thyroid cancer cells. Our study demonstrated that the *KDR* gene in thyroid cancer cells was almost completely knocked out, accompanied by the inhibition of cell growth suppression and the loss of invasion ability. This inhibitory effect suggested that KDR mediates VEGF signaling and plays a critical role in thyroid cancer development in cancer cell growth and cancer metastasis. Even though with the strong evidence showed in this study, there is still KDR targeted anti‐cancer drug approved by the in advanced thyroid cancer. Thus, developing effective and selective inhibitors or combinational drug treatments targeting KDR in advanced thyroid cancer patients remains unexplored.

## Conflict of interest

The authors declare no conflict of interest.

## Author contributions

MLT, CLL, and CHL designed the research experiments. WNL and KWH performed the experiments and acquired data. CYL and YHC helped design the CRISPR protospacers and advised on the virus production and infection procedures. AWL and CLL helped interpret the results and analyzed gene editing efficiency. MLT, LCH, YHC, CLL, and CHL participated in clinical discussion and drug indication. MLT, CLL, and CHL conceived the study and supervised the project. All authors read and approved the final manuscript.

## Supporting information


**Fig. S1**. Western blot membranes of Figure 2K.
**Fig. S2**. (A) Crystal violet stain image of Figure 3B. (B) Microscopy image of Figure 3C. (C) Western blot membrane of Figure 3E.
**Fig. S3**. Microscopy image of Figure 5A.
**Fig. S4**. (A) Western blot membranes of Figure 5C. (B) Western blot membranes of Figure 5D.

## Data Availability

The data used to support the findings of this study are available from the corresponding author upon request.
