# Supplementary material for: CRISPR‐mediated knockout of VEGFR2/KDR inhibits cell growth in a squamous thyroid cancer cell line
Source: FEBS Open Bio. 2022 Apr 8;12(5):993–1005. doi: 10.1002/2211-5463.13399 (PMC9063427; doi:10.1002/2211-5463.13399)

Supplementary figure 1

Western blot membranes of Figure 2K

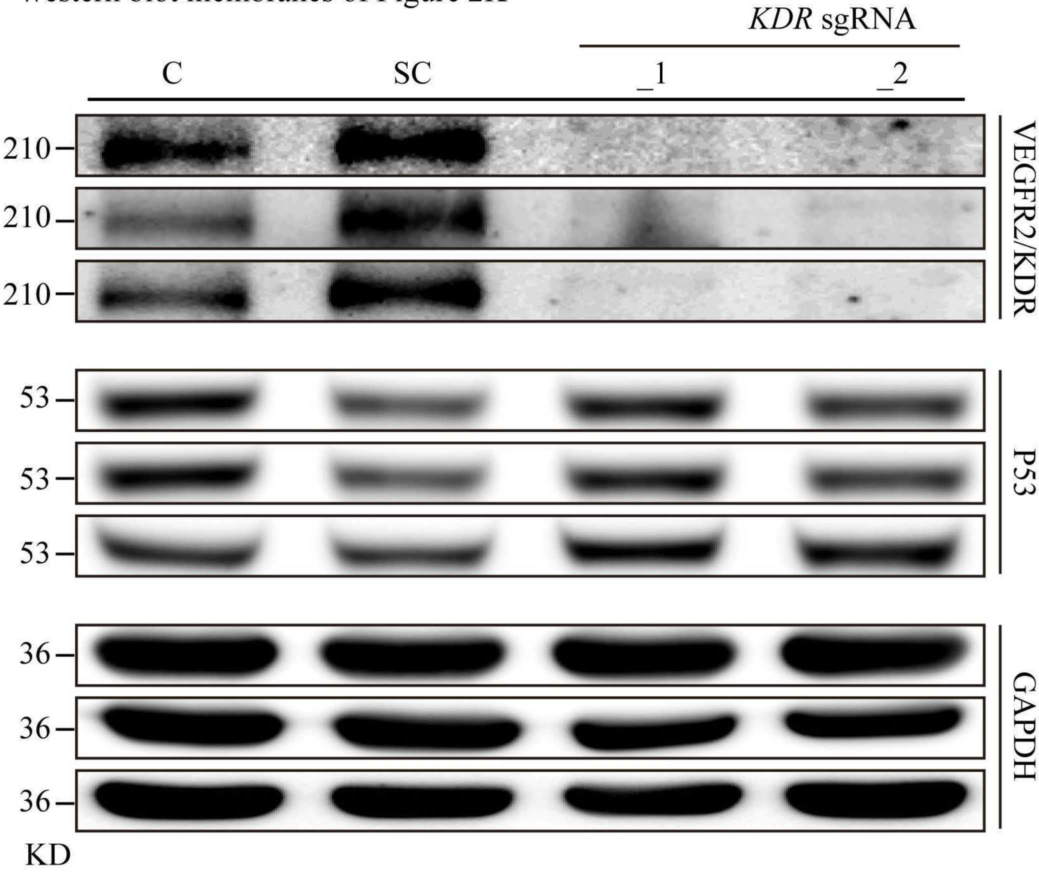

Supplementary figure 2

A

Crystal violet stain image of Figure 3B

SC

KDR sgRNA_2

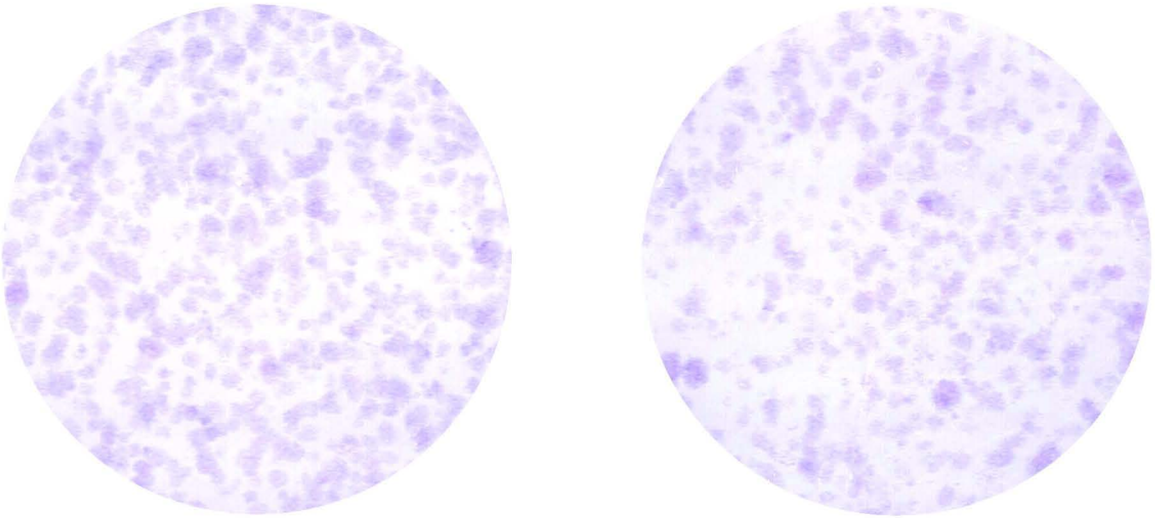

B

Microscopy image of Figure 3C

SC

KDR sgRNA_2

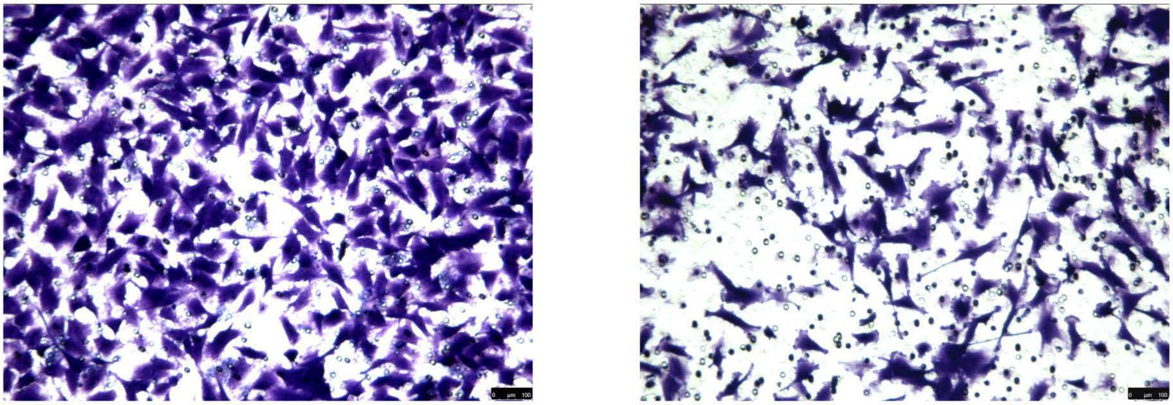

C

Western blot membrane of Figure 3E

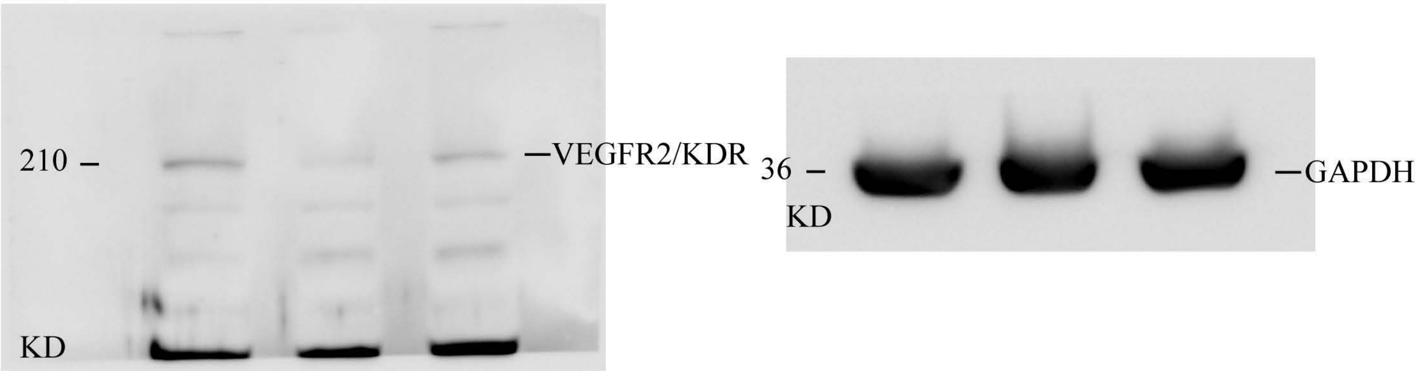

Supplementary figure 3

Original microscopy image of Figure 5A

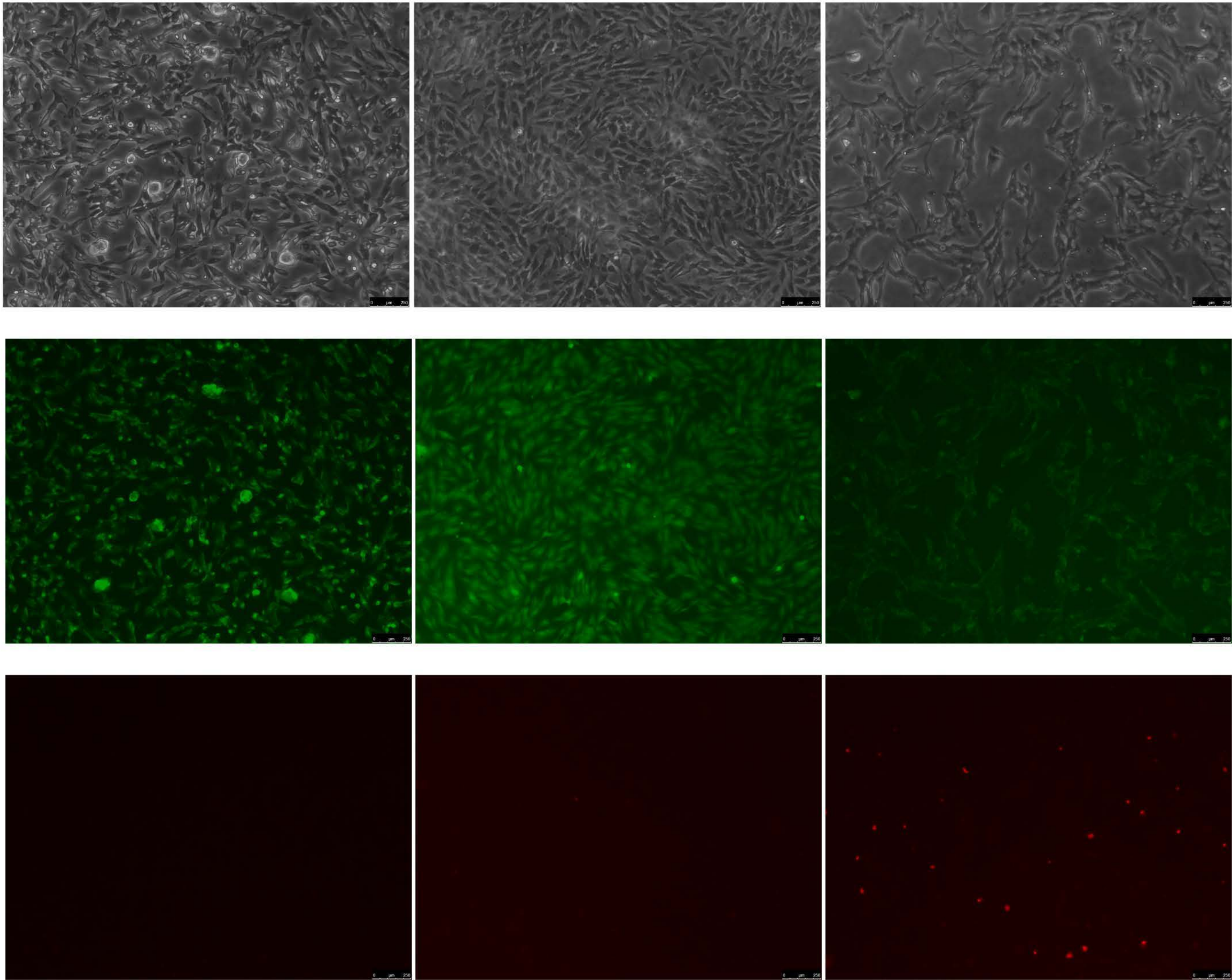

Supplementary figure 4

Western blot membranes of Figure 5C

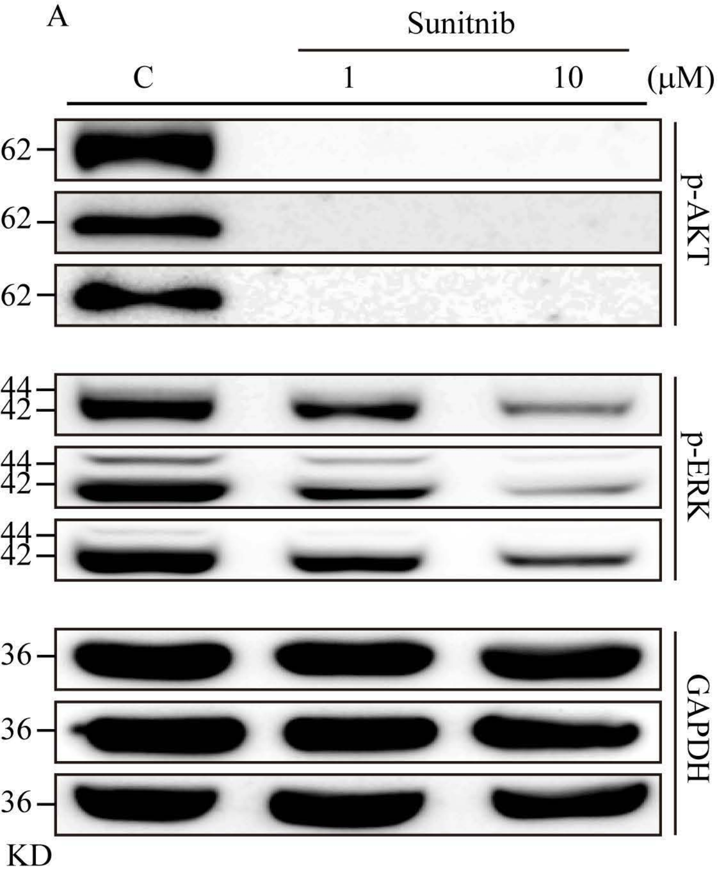

Western blot membranes of Figure 5D

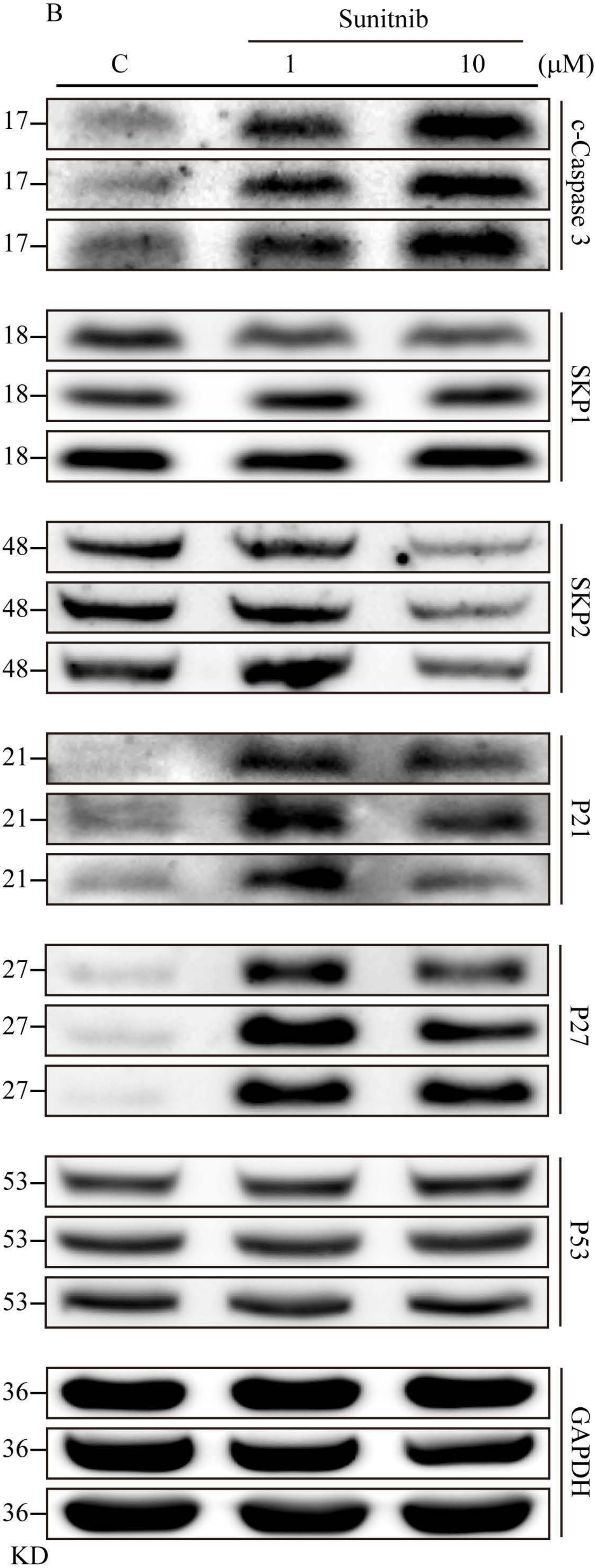

Supplement: Supplementary file 1 — Fig. S1. Western blot membranes of Figure 2K. Fig. S2. (A) Crystal violet stain image of Figure 3B. (B) Microscopy image of Figure 3C. (C) Western blot membrane of Figure 3E. Fig. S3. Microscopy image of Figure 5A. Fig. S4. (A) Western blot membranes of Figure 5C. (B) Western blot membranes of Figure 5D. [file FEB4-12-993-s001.pdf]
